# Supplementary material for: Prenatal Diagnosis of Talipes Equinovarus by Ultrasound and Chromosomal Microarray Analysis: A Chinese Single-Center Retrospective Study
Source: Genes (Basel). 2022 Sep 1;13(9):1573. doi: 10.3390/genes13091573 (PMC9498837; doi:10.3390/genes13091573)
Supplement: Supplementary file 1 [file genes-13-01573-s001.zip › genes-1848601-supplementary.pdf]

Supplementary Table S1. Clinical characteristics of TE fetuses with VOUS.

Case	Maternal Age (Years)	GA (Weeks)	Ultrasound Findings	CMA Results	Type of CNV	Size (Mb)	Outcome
**1**	23.4	26	Isolated TE	arr3q22.1(132298557-132647420) × 3<br>arr12q24.33(132026536-132278530) × 3	Duplication<br>Duplication	0.35<br>0.25	TOP
**2**	24.5	23.9	Isolated TE	arr5p15.33(987,177-1,226,807) × 3<br>arr20q11.21(29,652,121-29,917,507) × 3	Duplication<br>Duplication	0.24<br>0.27	Live birth
**3**	23.9	25.1	Isolated TE	arr1p36.33(1439390-1652893)×1	Deletion	0.21	Live birth
**4**	38.1	19.0	Isolated TE	arr17p11.2(16581523-16807265) × 3	Duplication	0.23	Live birth
**5**	29.2	24.2	Isolated TE	arr2q12.2q13(107,032,426-111,365,996) × 3	Duplication	4.33	Live birth
**6**	34.7	23.3	Isolated TE	arr4p14(36142523-36837111) × 3<br>arr18p11.31(4002199-4204376) × 1	Duplication Deletion	0.70<br>0.20	Live birth
**7**	29.8	24.1	Isolated TE	arr2q24.3(165,696,963-165,828,391) × 3	Duplication	0.13	Live birth
**8**	26.6.	22.1	Isolated TE	arr1q31.1q31.2(190226234-191271408) × 3	Duplication	1.05	Live birth
**9**	22.9	29.6	Isolated TE	arr14q13.1(34296645-34642068) × 3	Duplication	0.36	Live birth
**10**	28.7	32.1	Isolated TE	arr8p23.1(11279747-11591196) × 3	Duplication	0.31	Live birth
**11**	26.8	23.1	Isolated TE	arr11q14.3(89,501,939-90,086,182) × 4	Duplication	0.58	Live birth
**12**	33.5	22.4	Isolated TE	arr4p14(39,846,353-40,130,593) × 3	Duplication	0.28	Live birth
**13**	30.4	24.4	Isolated TE	arr5q12.1(59,212,189-59,494,303) × 1	Deletion	0.28	Live birth
**14**	34.2	24.0	Isolated TE	arr15q26.3(99,521,882-100,158,444) × 3	Duplication	0.64	TOP
**15**	29.8	24.9	Isolated TE	arr21q22.3(47028160-47533226) × 3	Duplication	0.51	Live birth
**16**	33.0	24.3	Isolated TE	arr8p23.2(3685301-5935671) × 4	Duplication	2.25	Live birth
**17**	37.1	25.9	Isolated TE	arr16q23.1(75,276,258-75,612,925) × 3	Deletion	0.27	TOP
**18**	24.9	25.4	Isolated TE	arrXp11.23(47,707,712-48,261,360) × 2	Duplication	0.55	TOP
**19**	28.7	24.1	Isolated TE	arr8q24.11(118,226,133-118,884,778) × 3	Duplication	0.66	Live birth
**20**	29.6	31.1	TE; widened CSP	arr5p13.2(33951000-34157068) × 1	Deletion	0.21	LFU
**21**	34.2	19.3	TE; spina bifida	arr2q12.2q12.3(106872677-108533926) × 3<br>arr3p25.2(12594564-12826919) × 3	Duplication<br>Duplication	1.66<br>0.32	TOP
**22**	30.8	31.3	TE; polyhydramnios	arr12q23.3(104,573,529-104,797,526) × 3	Duplication	0.22	TOP
**23**	37.8	24.7	TE; omphalocele	arr6q25.3(158,499,583-158,761,112) × 3	Duplication	0.26	TOP
**24**	29.3	24.4	TE; FGR	arr7p22.3(1976262-2246007) × 1	Deletion	0.27	TOP
**25**	27.7	13.3	TE; meningomyelocele	arr8q22.2(100137795-100557343) × 1	Deletion	0.42	TOP

TE: talipes equinovarus; GA: gestational age; CNVs: copy number variations; TOP: termination of pregnancy; CSP: cave of septum pellucidum; LFU: lost to follow-up; FGR: fetal growth restriction.
